# Role of endothelial glycocalyx in sliding friction at the catheter-blood vessel interface

**DOI:** 10.1038/s41598-020-68870-x

**Published:** 2020-07-16

**Authors:** Chengxiong Lin, Hans J. Kaper, Wei Li, Robert Splinter, Prashant Kumar Sharma

**Affiliations:** 1 Department of Biomedical Engineering, University of Groningen and University Medical Center Groningen, Groningen, The Netherlands; 2 Tribology Research Institute, Key Laboratory for Advanced Technology of Materials of Ministry of Education, Southwest Jiaotong University, Chengdu, 610031 China; 3 PendraCare, Wellinq, 9351 VC Leek, The Netherlands

**Keywords:** Cardiac device therapy, Biomedical materials

## Abstract

Catheterization is a common medical operation to diagnose and treat cardiovascular diseases. The blood vessel lumen is coated with endothelial glycocalyx layer (EGL), which is important for the permeability and diffusion through the blood vessels wall, blood hemodynamics and mechanotransduction. However EGL’s role in catheter-blood vessel friction is not explored. We use a porcine aorta to mimic the blood vessel and a catheter loop was made to rub in reciprocating sliding mode against it to understand the role of catheter loop curvature, stiffness, normal load, sliding speed and EGL on the friction properties. Trypsin treatment was used to cause a degradation of the EGL. Decrease in catheter loop stiffness and EGL degradation were the strongest factors which dramatically increased the coefficient of friction (COF) and frictional energy dissipation at the aorta-catheter interface. Increasing sliding speed caused an increase but increase in normal load first caused a decrease and then an increase in the COF and frictional energy. These results provide the basic data for safety of operation and damage control during catheterization in patients with degraded EGL.

## Introduction

Cardiovascular diseases (CVDs) are a group of disorders of the heart and blood vessels^1^. Although the incidence and the mortality rate of CVDs has decreased^2–5^, they still remain a leading cause of death all over the world according to the world health organization. In 2016, 17.9 million people died due to CVDs which was 31% of all global deaths^6^. The decline in CVD deaths is due to the health awareness and lifestyle changes but also due to the effective diagnosis and intervention. Minimally invasive transcatheter cardiovascular interventions are the most popular operations with about 200 million endovascular catheterizations taking place every year^7^. Often radial (forehand) or femoral (thigh) artery is chosen for catheter insertion. For the catheter to reach the vicinity of the heart, it commonly requires a catheter of 100 cm in length. Depending on the pathogenesis, a catheter may spend a few seconds to few days in vivo for diagnosis and treatment^8,9^. Both the insertion of catheter and intervention requires continuous sliding contact between the catheter and the lumen of the blood vessels.

A thin (~ 500 nm), gel-like endothelial glycocalyx layer (EGL) is present on the luminal side of the blood vessel. The EGL on the luminal surface of the aorta mainly consists of mesh of membrane bound proteoglycans e.g. syndecan-1 and glypican containing chondroitin sulfate and heparin sulfate side chains and secreted glycosaminoglycans like hyaluronic acid and associated plasma proteins^10–12^. The polyanionic nature of these molecules provides an overall negative charge to the EGL^11^ which plays an important role in protection, regulation, diffusion and antiadhesion^13,14^ on the luminal surface of blood vessel. The EGL is delicate and the degradation of glycocalyx structures was found to occur after provocation with inflammatory and atherogenic stimuli, such as TNF-α administration^15^, ischemia/reperfusion^16^, and infusion of oxidized low-density lipoprotein^17^. Studies concerning the glycocalyx damage in literature has been performed at different scales i.e. from single cells, isolated organs, whole animal models and clinical studies^12^. The change of dextrans has been used to monitor the disruption and shedding of the glycocalyx in vitro, for isolated organs and cells. The stimuli included TNF-$\alpha$, atrial natriuretic peptide, abnormal blood shear stress etc.^15,18,19^. As a contrast, hyperglycemia, hemorrhagic shock, inflammation and ischemia–reperfusion injury were found to be related to the disruption of the glycocalyx in animal models^20–22^. For clinical studies, the changes of four primary components of the glycocalyx (syndecan-1, heparan sulfate, hyaluronic acid and chondroitin sulfate) were observed due to hypovolemia, aortic or cardiac bypass surgery and endothelial cell damage^23–25^. The EGL shedding and degradation shows a great impact on the physiological function of soft tissue as well as the mechanical performance^14^. EGL shedding has been shown to effect the barrier function^26^ i.e. permeability and diffusion through the blood vessels wall^1^, blood hemodynamics^27^ and mechanotransduction^28^. Since catheterization is most likely be performed under health conditions leading to EGL degradation, it is surprising that not much attention has been paid to the effect of the EGL shedding and degradation on the sliding interaction between the catheter and the luminal blood vessel. Increase in friction at the catheter/blood vessel interface may cause serious damage of soft tissue owning to adhesion (leucocyte, protein etc.) and deformation of the catheter-blood vessel contact^29–31^. Therapeutic vascular catheterization techniques are sometimes hampered by the frictional forces between the blood vessel and catheter^32^, which can also induce vasoconstriction and injury and result in reactive intimal proliferation or distal embolization associated with end-organ ischemia and infarction^33,34^.

The role played by load, sliding speed, presence of EGL, catheter curvature on the frictional behavior of the blood vessel-catheter interface is not well described in the literature. Therefore, this is the main aim of this study and in order to achieve the aim an accurate and effective contact model between the blood vessel and vascular catheter has been developed.

Most of the catheters are coated with a lubricious films^35–42^ e.g. hydrophilic poly (vinyl pyrollidone) (PVP), poly (MPC-co-BMA) phospholipid polymer, ComfortCoat®^35^ etc. However, the distal, curved end is left uncoated to allow a better frictional feedback for the surgeon, which helps them position the catheter precisely. The distal end, in fact, slides for the longest distance in vivo to reach the location of intervention. Thus uncoated catheter is used for the tribology measurements in this study. We have used the luminal part of the porcine aorta to mimic the lumina of the blood vessels. First, we characterized the catheter in the form of a loop and catheter-aorta interface with respect to the loop stiffness and catheter-aorta contact area. Then the friction behavior of the aorta–catheter interface at different normal load, sliding velocity, catheter loop stiffness and EGL degradation was characterized. These results can provide the basic data for safety of operation and damage control during cardiovascular catheterization in patients with degraded EGL.

## Materials and methods

### Specimen preparation

The porcine aortas were chosen as the experiment sample in this study owning to the similarity in physiological structure and function to the human aorta^43^. The aortas were obtained from the local slaughterhouse (Kroon Vlees, Groningen 9723 TM, Netherlands). The weight of each pig was circa 90 kg and the age was about 3 months. The length of each intact aorta from the slaughterhouse was 18–22 cm and delivered to the laboratory within 2 h postmortem which stored in a ice box, and tested within 4 h after extraction so as to avoid dehydration. Firstly, the outside adventitia and the fat layer of aortas were removed with scalpel to ensure the smoothness of samples. Then aortas were cut along the central axis and the sample size was about 60 mm × 25 mm for friction test, and 5 mm × 5 mm for fluorescent microscopy. The thickness of the aorta was 1.5 to 3 mm. The experiment samples were finally washed with phosphate-buffered saline (PBS, pH 7.0) solution and then put in refrigerator (4 °C) with PBS solution before the test. The vascular catheter (*Pointer*, Wellinq, Groningen, The Netherlands) was devoid of a hydrophilic coating and had an outside diameter of 2.0 mm. All tests were performed in a laboratory at the temperature of 20–22 °C and submerges in PBS.

### Characterization of the catheter loop curvature, stiffness and catheter-aorta contact area

**Figure 1:**
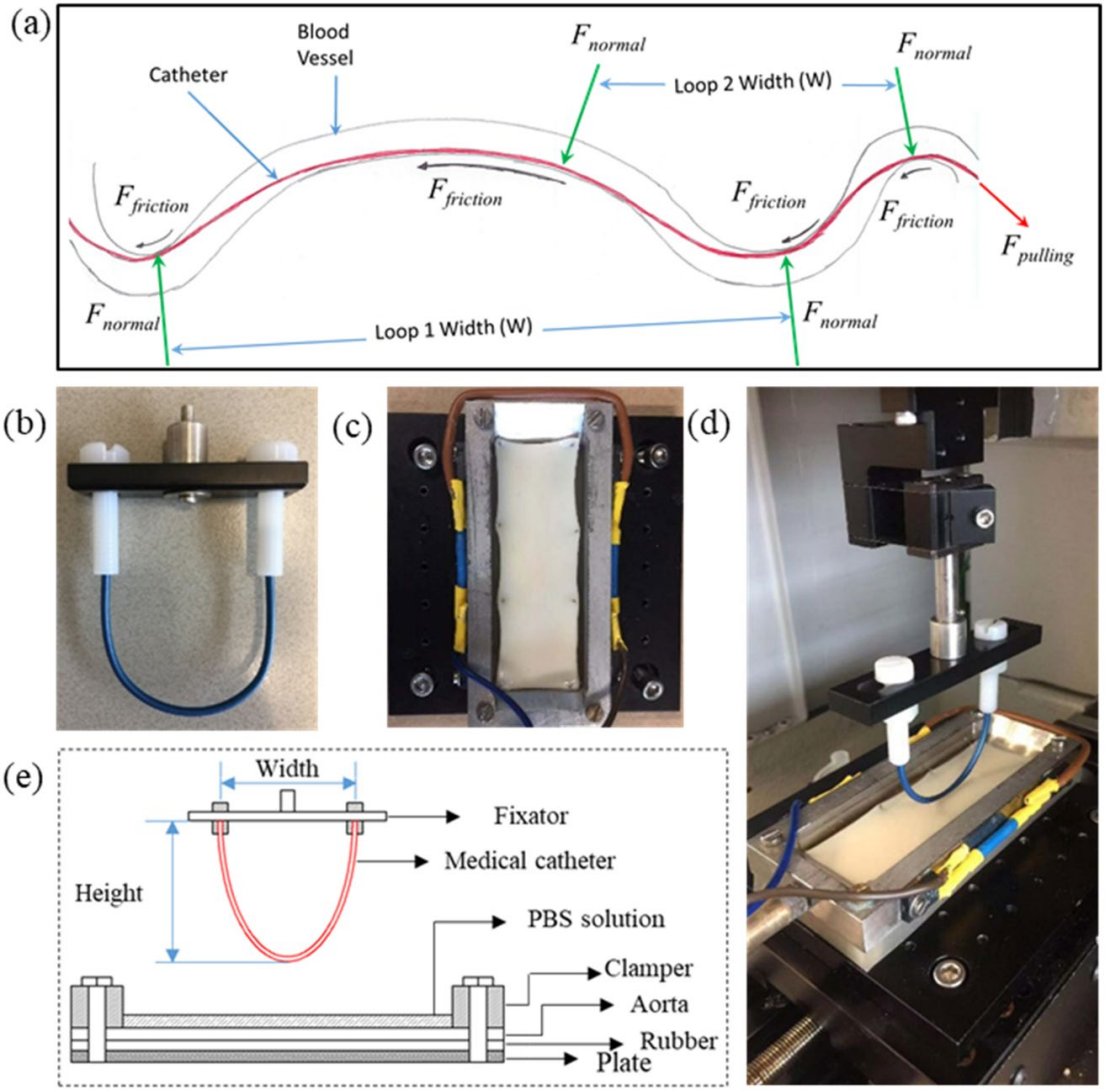
(**a**) The loops in 2D which the catheter needs to form in order to travel and slide inside a tortuous human blood vessel. Each loop has its own length (L) and width (W). The pulling force on the catheter gives rise to normal and friction force at each contact point. L relative to W gives rise to a certain loop curvature and stiffness. The model catheter loop prepared for testing (**b**), piece of aorta mounted on the rubber (**c**), sliding tribological pair (**d**) and schematic diagram of the test setup (**e**).

The blood vessels in vivo are 3-dimensionally curved, twisted and kinked and the deviation from linearity is quantified in terms of tortuosity^44^. The catheter needs to follow the tortuous blood vessels to reach the destination of diagnosis or treatment (Fig. 1a). In this study we used 2 dimensional catheter loops to measure the sliding friction, thus for simplicity we have quantified their curvature (mm^−1^), which is the inverse of the radius at the point of catheter-aorta contact. The model catheter loops for testing were prepared as shown in Fig. 1b where the length (L) of the catheter was varied from 10 to 22 cm in steps of 3 cm and the width (W) was kept constant at 3.90 cm, this gave rise to a L/W ratio of 2.55 to 5.61 The model catheter loops of different length were placed on a graph paper and photographed to calculate the curvature using Matlab 2018 (MathWorks, USA). The catheter loops and aorta were mounted on the UMT and gently brought in contact (Fig. 1d) with a preload force of 0.2 N and slowly increased to 0.75 N while monitoring the displacement of the catheter loop. The slope of load versus displacement was taken as the stiffness in N/mm. Then a pressure sensitive prescale film (4LW, Prescale, Fujifilm, Japan) was placed between the catheter loop and the porcine aorta, brought in contact and pressed again with 0.75 N to record the contact area. The pink contact region on white prescale film was photographed together with graph paper. A contour was drawn by hand around the pink impression and the corresponding contact area was calculated with Matlab 2018 (MathWorks, USA). All the test were repeated 5 times.

### Friction tests

As the catheter is pulled or pushed through the tortuous blood vessels with a certain force (*F*_*pulling*_) and speed, it gives rise to a normal force (*F*_*normal*_) at each bend of the loop (Fig. 1a). The normal force can be calculated according to the Euler-Eytelwein formulae^45^. In presence of a finite normal force a friction force will be generated at each contact point between the catheter and the blood vessel. Due to the complexity in predicting the in vivo normal force and sliding speeds the sliding friction was measured at a range of normal forces and sliding speeds. The contact between the catheter and the luminal surface of the blood vessel during cardiovascular catheterization was simulated in reciprocating sliding mode on a multi-specimen Biomedical Micro-Tribometer, Universal Mechanical tester-3 (UMT-3, Bruker Corporation, American). The prepared aorta sample was placed on the silicon rubber and fixed with pins around the boundary of the aorta (Fig. 1c,e) and mounted on the specially designed bath. The volume of the PBS solution in bath was 8 ml every time so that the liquid could immerse contact point between the catheter and aorta. The temperature of the PBS solution in bath was set to 37 °C to simulate the human internal environment. The support frame with the catheter loop (counterpart of aorta) was mounted to a suspension system attached to the load cell (Fig. 1d). During all initial tests, the catheter was pressed onto surface of the aorta at a programmed speed of 0.05 mm/s to preload. When the force reached the pre-set value, the catheter was slid linearly at a constant sliding speed. According to the suggestion of the medical practitioners as well as the manufacturer of catheter, the normal load was varied from 0.3 to 1.2 N in steps of 0.3 N, the sliding speed was varied from 2 to 10 mm/s in steps of 2 mm/s. Three different model catheters, named A, B and C, were used in the study. The only difference was their length i.e. catheter A, B and C here were 112.11 mm, 127.24 mm and 144.25 mm long respectively. Friction force was measured for 10 reciprocating cycles with a sliding distance of 60 mm per cycle using the UMT-3 tribometer at a sampling rate of 20 kHz. Measured friction force was divided by the applied normal force to calculate the coefficient of friction (COF). While sliding back and forth a friction loop was observed (Fig. S2) for each cycle and the area inside the loop gave the amount of energy dissipated in each loop. Besides the COF, frictional energy dissipation was also quantified. The room temperature is 20–22 °C and the humidity is 50–70%.

### Glycocalyx (EGL) visualization and quantification using fluorescent microscopy

The EGL on the aorta surface was observed using the Confocal laser scanning microscopy (CLSM, Leica TCS SP2 Leica, Wetzlar, Germany) with an HCX APO L40 × /0.80 WU-V-1 objective. Fluorescent stain Con A (Concanavalin A, Alexa Fluor™ 488 Conjugate from ThermoFisher, Catalog no. C11252) was used to stain the EGL and 6-diamidino-2-phenylindole (DAPI, CAS Number 28718–90-3, Sigma-Aldrich) for the nucleus of the endothelial cells. An argon ion laser at 488 nm and a green HeNe laser at 543 nm were used to excite Con A and DAPI respectively. The fluorescent signal was collected between 500 and 540 nm for Con A and rendered green while the signal collected between 583–688 nm for DAPI was rendered blue. The EGL on the luminal surface of the aorta mainly consists of mesh of membrane bound proteoglycans e.g. syndecan-1 and glypican containing chondroitin sulfate and heparin sulfate side chains and secreted glycosaminoglycans like hyaluronic acid and associated plasma proteins^10–12^. Trypsin (Trypsin, CAS Number 9002-07-7, Sigma-Aldrich) was chosen to degrade the glycocalyx^46^. The trypsin powder was dissolved in PBS solution (pH = 7.4) and 1.5% bovine serum albumin (BSA, Sigma-Aldrich). The size of sample for fluorescence test was 5 mm × 5 mm which was placed in the 24 well cell culture plate. Each piece of aorta was submerged in 1 ml of trypsin solution for different time durations (1 h or 24 h) and concentrations (125 μg/ml or 1,250 μg/ml). Then the enzyme solution was removed with pipette and the samples were washed thrice for 5 min with PBS solution. Aorta pieces were submerged in PBS and 1.5% BSA for the same amount of time as negative control. In order to minimize the biological variation adjacent pieces of the same aorta were used for trypsin treatment and negative control. Later paired statistics was used to determine the significance of trypsin treatment for EGL degradation. Afterwards, all samples were fixed with 3.7% paraformaldehyde (PFA) for 1 h and washed thrice for 5 min with PBS solution. Finally, adding 1 ml of PBS solution with ConA (5 μg/ml) and DAPI (4 μg/ml) to label the sample for 1 h in dark environment. CLSM was used to take fluorescent images, where the excitation laser intensity was kept same consistently for all aorta pieces. Finally, by setting the threshold, the fluorescence intensity was calculated with the ImageJ 1.50b software (Wayne Rasband, National Institutes of Health, USA)^47^.

### Statistics

The experimental data was presented as the mean value and standard deviation. F-test (analysis of variance) was used to determine the significant difference among different aorta samples under the same test conditions. The level of statistical significance was set to *p* < 0.05.

## Results

### Relation between the catheter loop curvature, stiffness and catheter-aorta contact area

**Figure 2:**
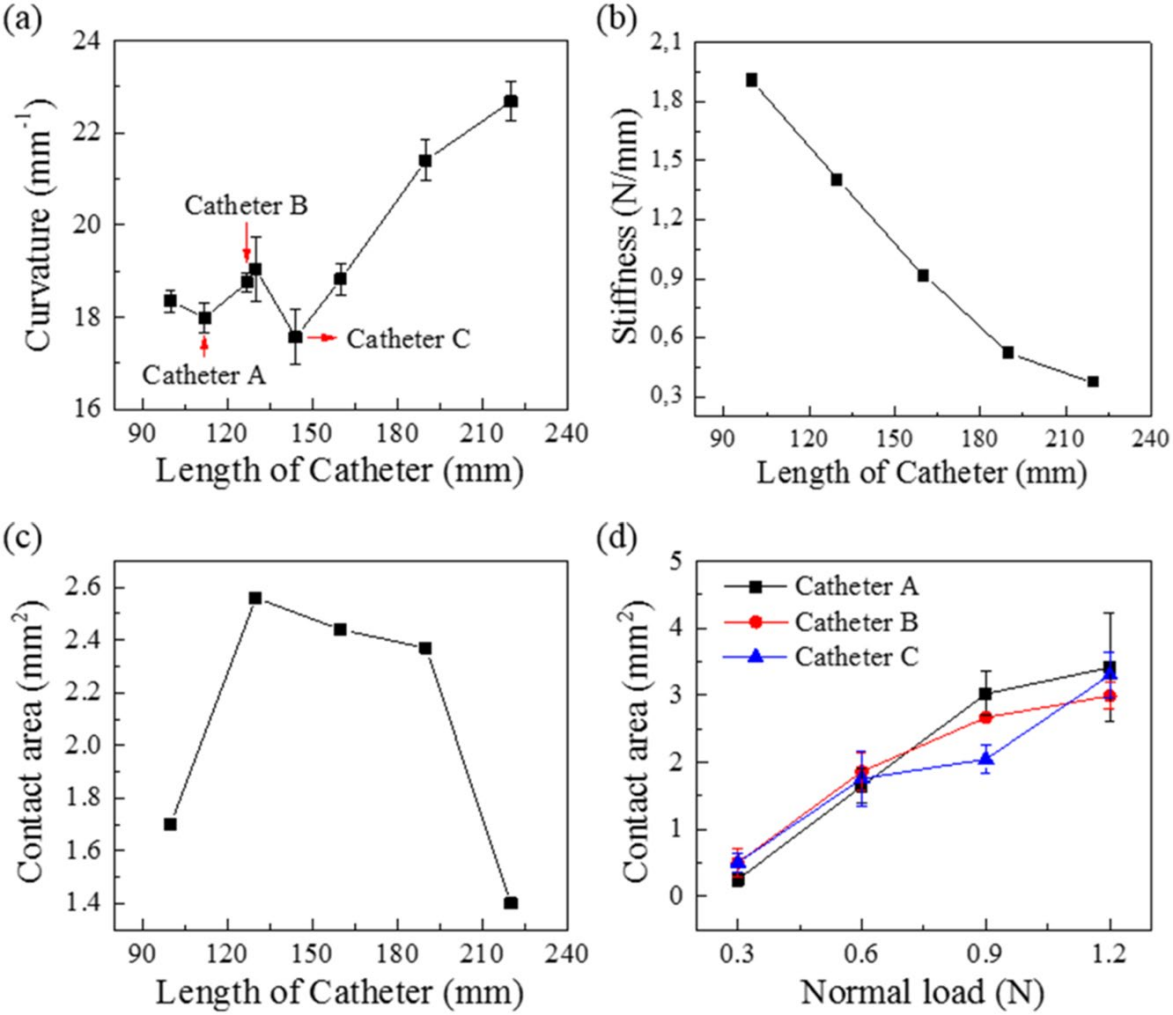
Physical and mechanical characterization of the model catheter loop and the catheter loop-aorta interface. Change in loop curvature (**a**) as a function of catheter loop with a constant width of 3.90 mm. The stiffness (**b**) and catheter-aorta contact area (**c**) at 0.75 N with increasing catheter length. And (**d**) the contact area change for model catheters A, B and C used for tribology testing as a function of the normal load. The error bars are standard deviation from 3 replicates.

As model catheter length increase from 100 to 160 mm at the constant width of 39.20 mm, the curvature remains more or less the same i.e. 18.33 ± 0.59 mm^−1^. But at model catheter lengths from 160 to 220 mm the curvature linearly increase from 18.82 to 22.68 mm^−1^ (Fig. 2a). The stiffness of model catheter loop decreases continuously from 0.92 to 0.37 N/mm as the length of the catheter increases (*p* < 0.05) (Fig. 2b). The contact area measured at 0.75 N first increased but then showed a decrease as the length of the catheter increased (Fig. 2c), this could be due to the combined effect of the continuous decrease in stiffness of the model catheter loop with length and the sensitivity limit of prescale i.e. when the stiffness becomes too low the prescale is not able to register any contact area. Model catheter loops A, B and C, when brought in contact with aorta show a continuous increase in contact area (Fig. 2d) with increasing the normal load. The only significant difference between the three model catheter loops is found at 0.9 N otherwise the contact area for the 3 catheters is very similar at other normal loads.

### The friction behavior of the aorta-catheter interface

Both the COF and frictional energy dissipated during catheter-aorta sliding was effected by the applied normal load, sliding speed and the catheter loop stiffness. In general an increase in normal force and sliding speed caused an increase in the COF and friction energy dissipation. On the other hand increase in catheter loop stiffness actually decreased the COF and friction energy dissipation. Detailed results are presented in the supplementary information and figures S1 and S3.

### Fluorescence intensity to quantify the endothelial glycocalyx layer (EGL) and its degradation

**Figure 3:**
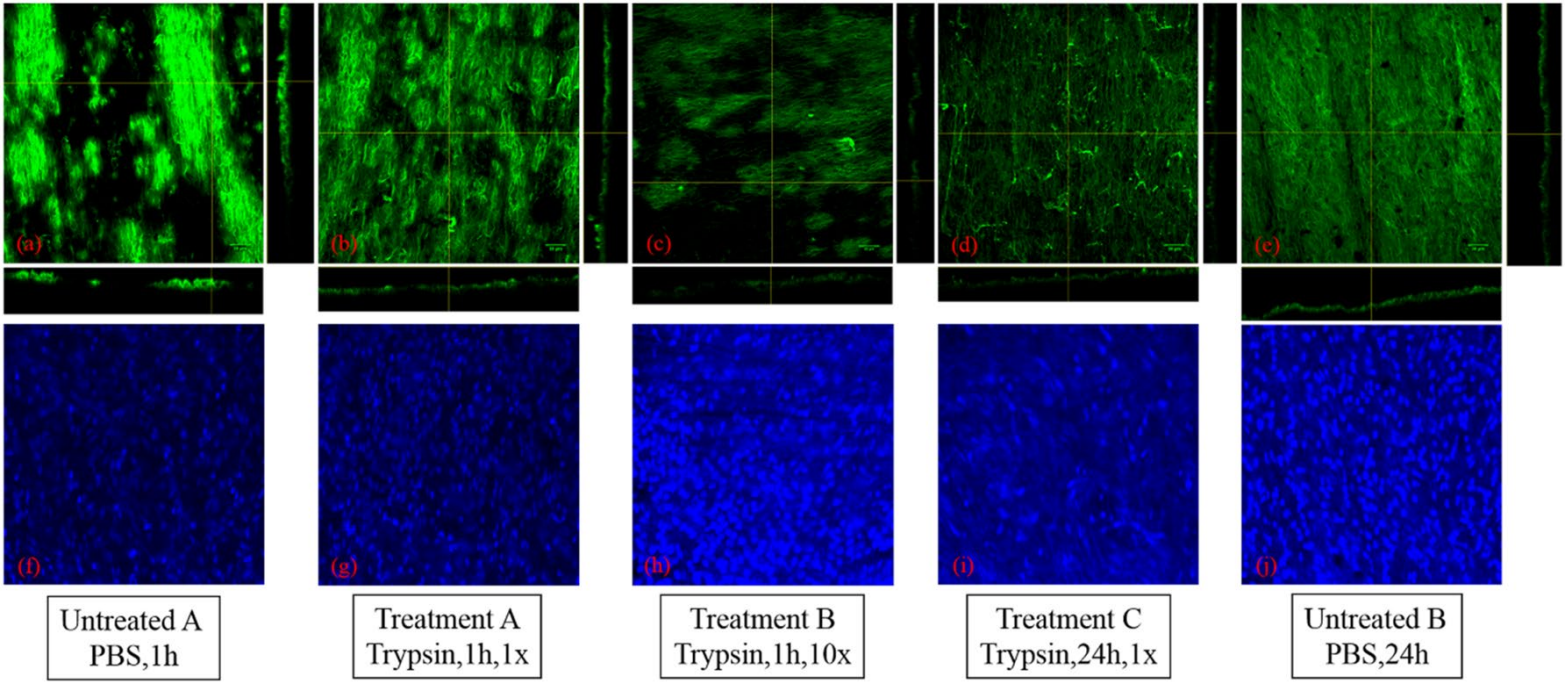
Confocal laser scanning fluorescence images of the luminal surface of the aorta after staining with Con A-Alexa and DAPI. Staining was performed after exposing the aorta surface to PBS for 1 h (**a**) and (**f**), 1 × trypsin for 1 h (**b**) and (**g**), 10 × trypsin for 1 h (**c**) and (**h**), 1 × trypsin for 24 h (**d**) and (**i**) and PBS for 24 h (**e**) and (**j**). 1 × is 125 μg/ml and 10 × is 1,250 μg/ml trypsin treatment.

**Figure 4:**
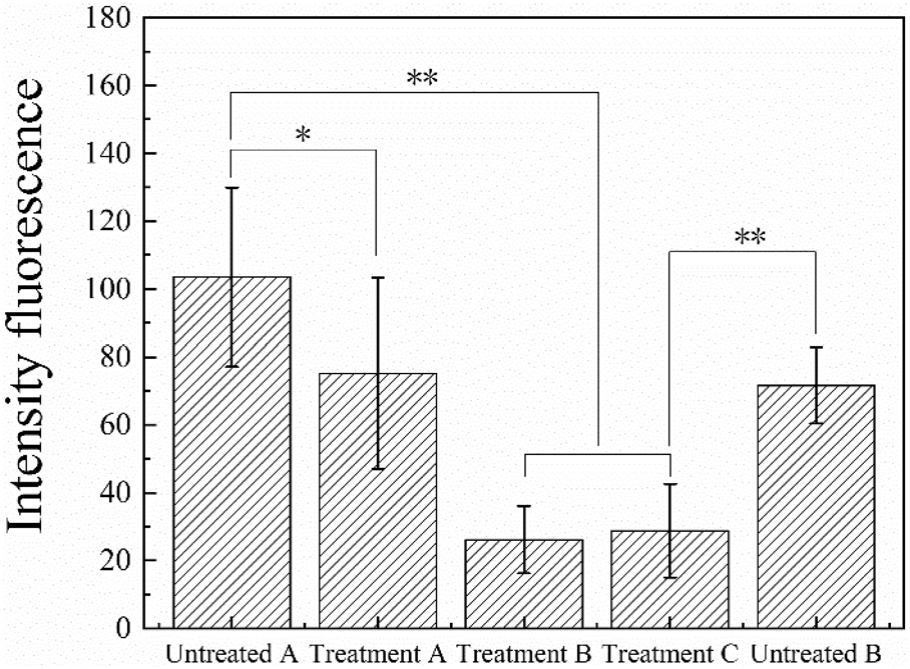
The fluorescent intensity on the surface of aorta under different experiment conditions: time, concentration and control. The x-axis labels correspond to Fig. 3. *means *p* < 0.05 and **means *p* < 0.01.

Figure 3 is the typical surface fluorescent images of the luminal surface of the aorta after staining with con A and DAPI. The former was used for labelling the EGL and the latter for labelling the nucleus of the endothelial cell lining. Each figure in Fig. 3 is a stack of layers owning to the surface roughness and the thickness of each layer is 2 µm. In order to obtain complete and effective data, there were at least three acquisition positions for each fluorescent sample and the average result of fluorescent intensity is shown in Fig. 4. The fluorescent intensity shows difference between the samples with treatment and control from Fig. 4a–e. All samples have clear nucleus under different treatment. The control sample presents the brightest color compared with other four pictures, namely the content of protein. As a contrast, the trypsin has better effect on samples under treatment (1 × 24 h, 10 × 1 h). Besides, the orthotropic section of each figures also present a certain difference, the EGL in Fig. 3a seems to be the thickest and the most obvious.

Trypsin was used to remove the EGL and the fluorescent intensity of the con A was measured to quantify the amount of EGL remaining on the aorta surface. Figure 4 presents the difference between the control (1 h, PBS solution) and the treatments (1 h, 125 μg/ml trypsin) (*p* < 0.05). There is a significant difference between the control (1 h, PBS solution) and the treatments (1 h 1,250 μg/ml trypsin and 24 h 125 μg/ml trypsin) (*p* < 0.01), which illustrates that the concentration of trypsin and the duration of treatment both affect the removal of EGL. The fluorescent intensity of the sample for 24 h control (24 h, PBS solution) is lower than the sample for 1 h control (1 h, PBS solution). Both Figs. 3 and 4 show that EGL was degraded with the trypsin treatment, although complete removal does not take place. If we take the fluorescent intensity as the measure of the amount of EGL left then we can see that the remaining EGL after 1 h 125 μg/ml, 1 h 1,250 μg/ml and 24 h 125 μg/ml trypsin treatment was 72.6%, 25.3% and 27.9% respectively.

### Change in the catheter-aorta friction after trypsin treatment

**Figure 5:**
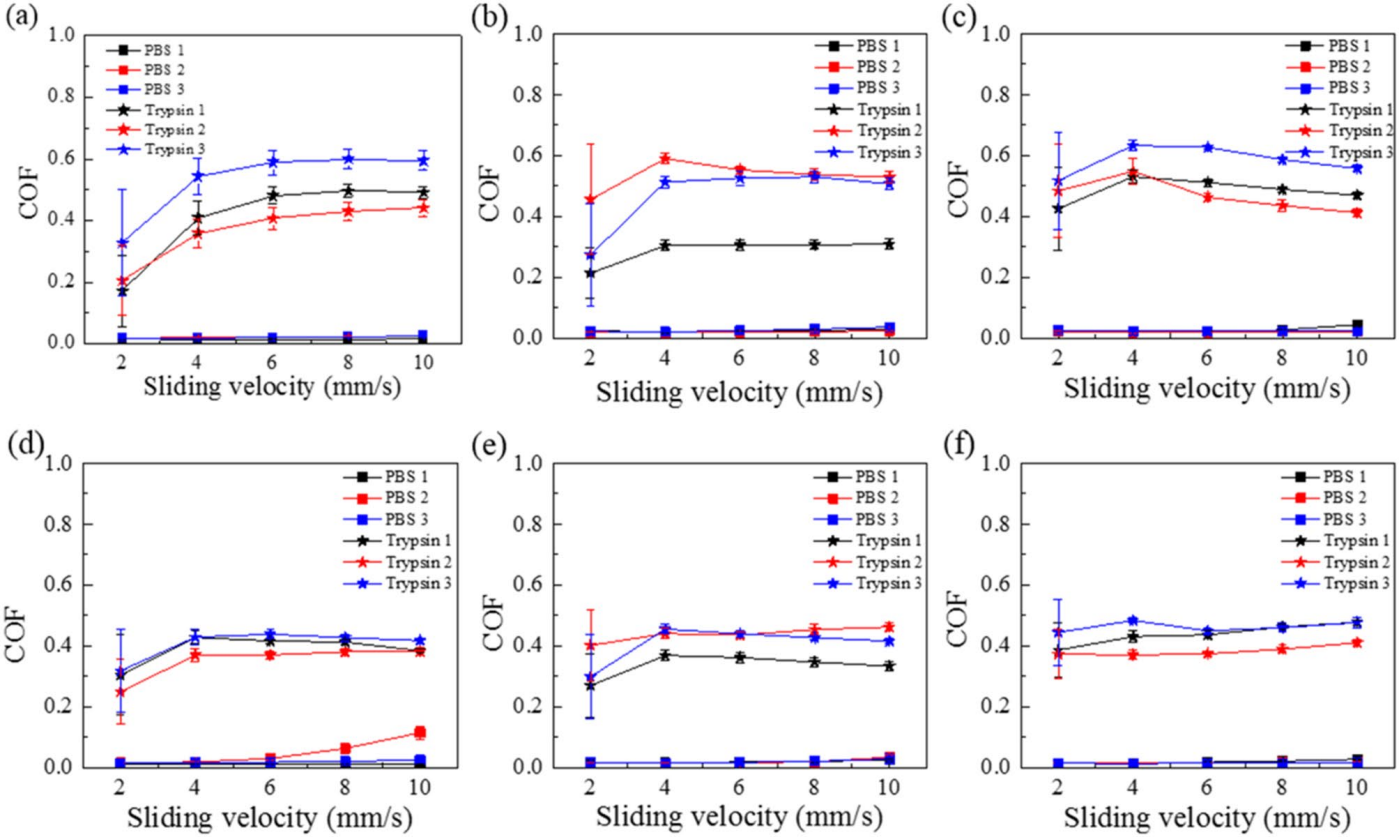
Triplicate measurements of friction measured using catheter A on aorta pieces either with PBS or trypsin at two different loads as a function of sliding speed. In order to keep the biological variations to a minimum, two adjacent pieces from each aorta were submerged in PBS and trypsin. The PBS 1& Trypsin1, PBS 2& Trypsin2 and PBS 3& Trypsin3 all represent the three repeatable measurement (three samples) in one experiment. e.g. PBS1 and Trypsin1 treatments are performed on 2 adjacent pieces obtained from the same aorta. Post treatment friction results are shown in the same color but with different symbols: (**a**) and (**d**) are at 0.3 N and 0.9 N respectively with 1 h trypsin treatment (125 μg/ml), (**b**) and (**e**) are at 0.3 N and 0.9 N respectively with 1 h trypsin treatment (1,250 μg/ml), (**c**) and (**f**) are at 0.3 N and 0.9 N respectively with 24 h trypsin treatment (125 μg/ml). (*p* < 0.01).

The friction measurements with catheter A (stiffness = 0.2 N/mm) show clear increase in COF after trypsin treatment Fig. 5. For the control sample with PBS solution, the COF section remain between 0.02 and 0.04 which is similar to results presented in Fig. S1. As a contrast, the COF is between 0.35 and 0.6 under the normal load of 0.3 N and 0.35–0.45 under the normal load of 0.9 N, which is more than ten times higher than control. Combined with the results of Figs. 3, 4 and 5, it also describe from side that the trypsin under different durations and concentrations has an effect on the lubrication environment at the catheter-aorta interface.

## Discussion

In this study we established a vascular catheter-blood vessel sliding friction model, using porcine aorta, for a better understanding of the role of catheter loop stiffness, sliding speed, normal load and most importantly the function of glycocalyx on the interfacial friction. Decrease in catheter loop stiffness and EGL degradation greatly affected the interfacial friction causing an order of magnitude increase in COF.

Due to the tortuous nature of the blood vessels, the catheter need to twist and turn in 3 dimensions while sliding inside the blood vessel (Fig. 1a). Increasing the length (L) of the model catheter while keeping the width (W) constant, i.e. increasing the L/W ratio, increases the curvature (mm^−1^) and decreases stiffness in N/mm (Fig. 2a,b). Although we have not explored this possibility but while keeping the L/W constant if the L and W are changed the curvature and stiffness might change. Furthermore, at lower stiffness the COF and frictional energy increases dramatically (Fig. S1 and S3 g, h, i, j). Thus in vivo we can expect that with lower tortuosity of the blood vessel i.e. loop 1 compared to loop 2 in Fig. 1a, would give rise to higher COF and friction energy dissipation. This is counter-intuitive but the reason is probably due to an increase in contact area with increasing L/W ratio. Due to the sensitivity limitations we were not able to register a continued increase in contact area as the L increased in Fig. 2c.

Three factors (applied normal load, sliding velocity and catheter loop stiffness) are affecting the frictional behavior at the aorta-catheter sliding interface (Fig. S1 and S3). The negative correlation between the COF and normal load was a commonly observed phenomenon for soft tissue, such as skin^48,49^, intestine^50,51^ and esophagus^30,52^. It’s mainly due to soft, irregular and rugged nature of the tissue surface which is rich in water and other liquids e.g. villus in the intestine and epithelial layer in the esophagus. When the normal load increased, more liquid can be squeezed, which can lubricate the interface to some extent as observed for catheter loop A (Fig. S1(a)). But at higher normal loads more and more liquid was squeezed out of the interface and the contact area increases due to deformation of the rough and rugged areas giving rise to an increase in friction. When the catheter slid through the curved blood vessels, the pulling or pushing force on the catheter would result in both normal and friction force between the catheter and the blood vessel.

When the catheter slid on the surface of aorta, the softer (less stiff) catheter can trigger bigger contact area between the vascular catheter and aorta as shown in Fig. 2. Meantime, the contact status changed from sliding friction into adhesion friction when the softer catheter slid under a bigger normal load and sliding velocity. On the other hand, when the normal load and sliding velocity both increased to some degree, the contact shape changed, it appeared as an arc instead of linear-shape duo to the instable contact between catheter and aorta. So the COF increases sharply as shown in Fig. S1(b) and (c). For the velocity dependence shown in Fig. S1(d-f), it’s also a common trend for friction model of soft tissue^52–54^. It’s mainly because deformation and recovery along with energy dissipation when the catheter sliding on the surface of aorta. At a lower speed, most energy of deformation recovered due to a finite loss of hysteresis friction. When the velocity increased gradually, the effect of stress relaxation is less obvious, which resulted in incremental hysteresis friction, namely energy dissipation. Thus, the increase of friction coefficient is inevitable. Increase in COF with sliding speed also indicates that any hydrodynamic or electrohydrodynamic mechanism of lubrication was not active but purely boundary lubrication at the aorta-catheter loop interface^55^.

Since the two sliding surfaces displace under the action of lateral friction force a certain amount of work needs to be done to overcome the frictional energy which gets dissipated^31^. With the increasing normal load and sliding velocity, the energy dissipation for catheter-aorta contact model both intensifies from Fig. S3. The bigger normal load can lead to bigger frictional force which meant greater energy dissipation. Meantime, according to the classical two-term biological theory^56,57^, assuming that the total of friction coefficient was composed of the sum of two independent contributions due to adhesion and deformation, respectively. As shown in Fig. S2, the contact stages changes from deformation friction to adhesion friction with the increasing normal load which results into bigger area of friction-displacement curve, namely bigger energy dissipation. In addition, just mentioned above, when the sliding velocity increased gradually, the effect of stress relaxation was less obvious and the aorta was hard to react in time, which resulted in incremental hysteresis friction, that’s energy dissipation. Considering the relationship between energy dissipation and damage of soft tissue^31^, it’s important to choose the contact stress and sliding velocity when the catheter contact the aorta during the cardiovascular surgery.

The surface of endothelial cells of aorta is decorated with a wide variety of membrane-bound macromolecules that constitute the glycocalyx. The glycocalyx consists of mesh of membranous glycoproteins, proteoglycans, glycosaminoglycans and associated plasma proteins^10^. As a result, we can find there is a clear effect of trypsin on destroying the glycocalyx by degrading the main protein contracture as shown in Figs. 3 and 4, especially for samples with concentration of 125 μg/ml (24 h) and 1,250 μg/ml (1 h). As mentioned in Introduction, the glycocalyx plays a role in protection, regulation and diffusion. Depletion of heparan sulfate (HS) and hyaluronic acid (HA), shear-induced NO production^28^ and removal of HS may cause a collapse of the glycocalyx which causes decreases in EGL thickness^1^. In current study, the friction behavior at the catheter-aorta interface presented a big difference before and after the treatment of trypsin as shown in Fig. 5. It illustrated for the first time from the side that the glycocalyx also had an effect on the lubrication at the catheter-aorta interface. As we know, there are some illnesses that can have an effect on or destroy the glycocalyx of aorta, such as inflammation, hypertension and so on. As a result, it’s should draw attention for medical workers that the cardiovascular catheterization for these patient can aggravate the damage of aorta.

Trypsin treatment protocol used in this study did not cause complete removal of EGL, 25.3% of the EGL was still remaining. Thus a ~ 75% reduction of EGL caused dramatic changes in the friction status i.e. tenfolds increase in coefficient of friction. This increase in coefficient of friction and friction energy dissipation could lead to arterial spasm^58^ or wear and tear of the epithelial cell lining on the luminal surface of the blood vessel. EGL degradation using heparinase (HS), chondroitinase ABC and hyaluronidase^1,59^, must be tested using the proposed protocol. This will help understand the contribution of each constituent of the EGL on the blood vessel-catheter friction and avoid the risk of endothelial cell removal due to trypsin treatment.

## Conclusions

A porcine aorta-uncoated catheter friction model was established, which helped understand the role played by the catheter loop stiffness, normal load, sliding speed and presence of EGL on the coefficient of friction and friction energy dissipation at the blood vessel-catheter interface.

1. The length (L) to width (W) ratio of the catheter loop was proportional to the curvature (mm^−1^) and inversely proportional to the stiffness (N/mm). L/W ratio also caused an increase in the catheter-aorta contact area, although the sensitivity of the film used for contact area measurement limited to its use to lower L/W ratios i.e. to relatively stiffer catheter loops. The contact area did show a clear increase with applied normal load.
2. The aorta-catheter COF and friction energy gradually decreased and then increased with the increasing normal load with an inflection point. The COF and frictional energy increased significantly with sliding speed. A decrease in catheter loop stiffness caused an order of magnitude increase in COF and friction energy.
3. EGL degradation through trypsin treatment caused an order of magnitude increase in the aorta-catheter COF and friction energy dissipation, which can lead to enhanced blood vessel spasm or wear and tear on the luminal side of the blood vessels.

## Supplementary information

Supplementary Information.
