# Supplementary material for: Role of endothelial glycocalyx in sliding friction at the catheter-blood vessel interface
Source: Sci Rep. 2020 Jul 16;10:11855. doi: 10.1038/s41598-020-68870-x (PMC7366638; doi:10.1038/s41598-020-68870-x)
Supplement: Supplementary file 1 — Supplementary Information. [file 41598_2020_68870_MOESM1_ESM.docx]

Supplementary Information

**Role of endothelial glycocalyx in sliding friction at the catheter-blood vessel interface**

**Chengxiong Lin^1,2^, Hans Kaper^1^, Wei Li^2^, Robert Splinter^3^, Prashant Sharma^1*^**

**3 Results:**

**3.5 The friction behavior of the aorta-catheter interface**

The normal force in the UMT-3 was set at 0.3 to 1.2 N with the steps of 0.3N. In the UMT-3 the up/down carriage movement tried to keep the normal force close to the set value. Furthermore, aorta was pinned on top of a 1cm thick silicone rubber sheet so that the undulations in aorta wall thickness can be smoothened out. Still Fig. S4 shows that fluctuations in normal force are not complete smoothened out. The coefficient of friction (COF) was calculated by dividing the measured friction force by the applied normal force at each time point at a frequency of 20Hz. The COF gradually decreases and then increases with the increasing normal load as shown in Fig. S1 (a, b, c) (p<0.05). The inflection point (point where the decreasing trend in COF transforms into the increasing trend) for catheter loop A moves towards lower applied normal loads at increasing sliding velocities, whereas for catheter loops B and C they remain independent of sliding speed. The COF is significantly related with the increasing sliding velocity for all the catheters from Fig. S1 (d, e, f) (p<0.05). The COF decreases by orders of magnitude with increasing stiffness (Fig. S1 g, h, i, j).


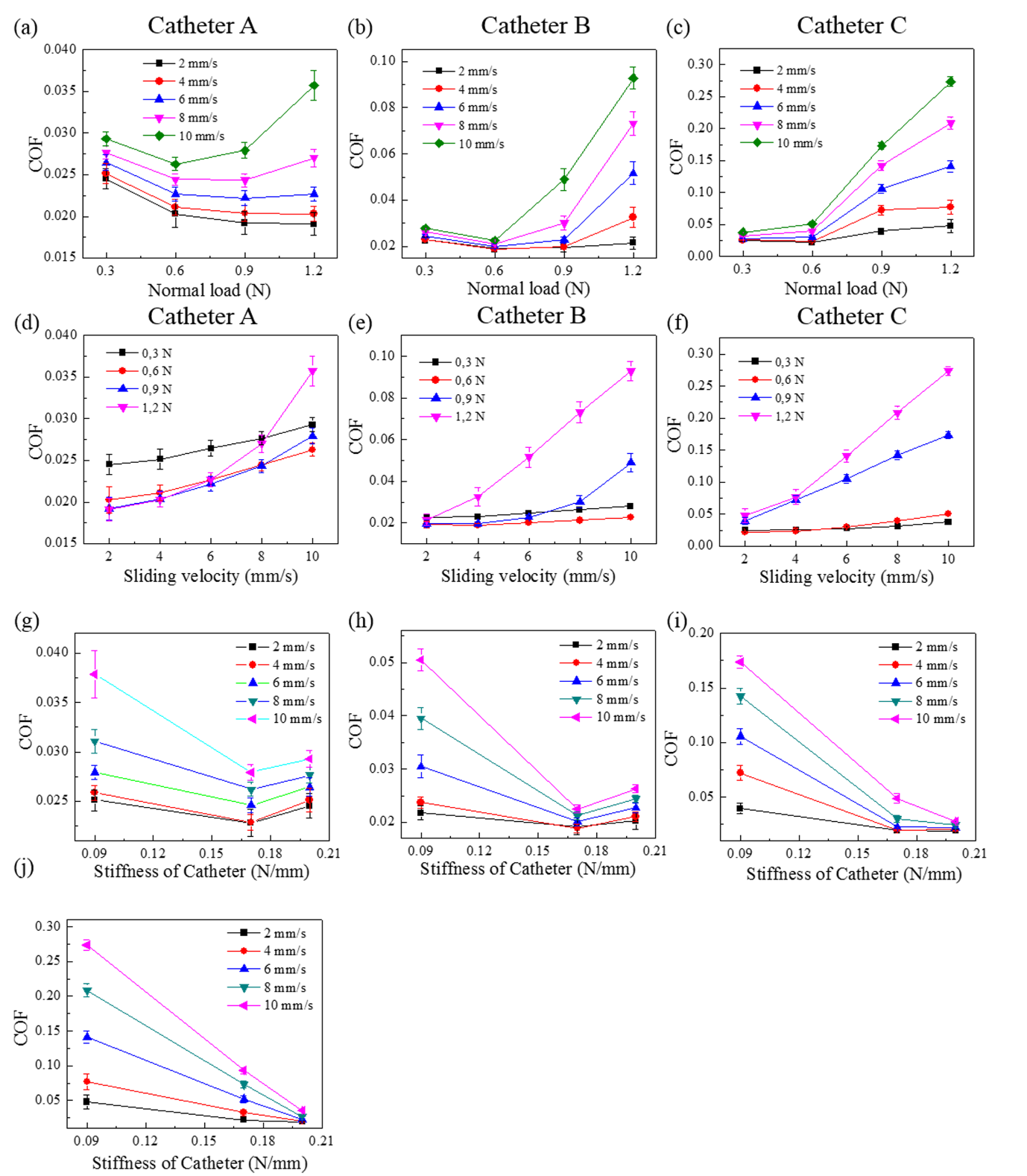


Fig. S1 The coefficient of friction for a sliding distance of 30 mm at the catheter-aorta interface under different normal loads (a, b, c), sliding speed (d, e, f) for catheter A, B and C respectively and catheter stiffness at different normal loads (g: 0.3 N, h: 0.6 N, i: 0.9 N, j: 1.2 N). Error bars are the standard deviations calculated from 5 replicate measurements.

For example, at a normal load of 1.2 N and sliding velocity of 10 mm/s the COF decreases from 0.275±0.007 to 0.093±0.004 to 0.033±0.001 for catheter loops C, B and A with stiffness of 0.09, 0.18 and 0.2 N/mm respectively. With the decreasing stiffness, the value of COF rapidly increases, especially for the curve of 1.2 N. For catheter A, the inflection point is 6 mm/s for the normal load of 1.2 N and 8 mm/s for the normal load of 0.9 N (Fig. S1 d). For catheter B and C, the curve of 1.2 N emerges a sharp difference relative to other curves. The results strongly indicate that three external factors (applied normal load, sliding velocity and catheter loop stiffness) affect the frictional behavior of the aorta and catheter sliding interface. From Fig. S1 g-j, there are clear difference between different catheter loops, namely difference stiffness. Especially for larger normal load, the tendency of COF presents negative correlation with the stiffness of the catheter (p<0.05).


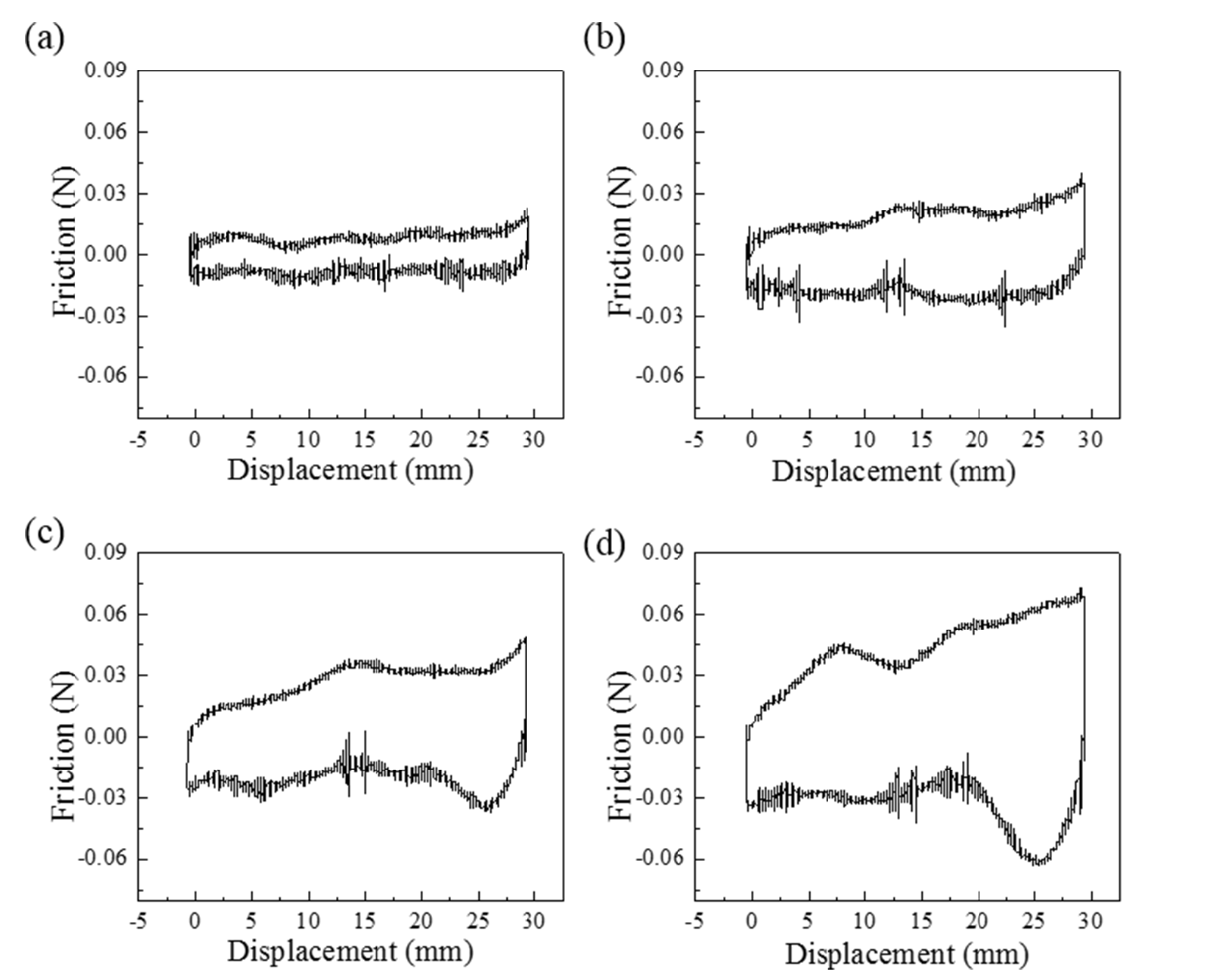


Fig. S2 Increasing hysteresis in the friction force versus displacement curve of catheter A at different normal loads (a) 0.3 N; (b) 0.6 N; (c) 0.9 N; (d) 1.2 N. Area inside each curve gives us the frictional energy dissipated per cycle. The upper curve corresponds the forward direction (positive value) and the nether curve corresponds the backward direction (negative value).


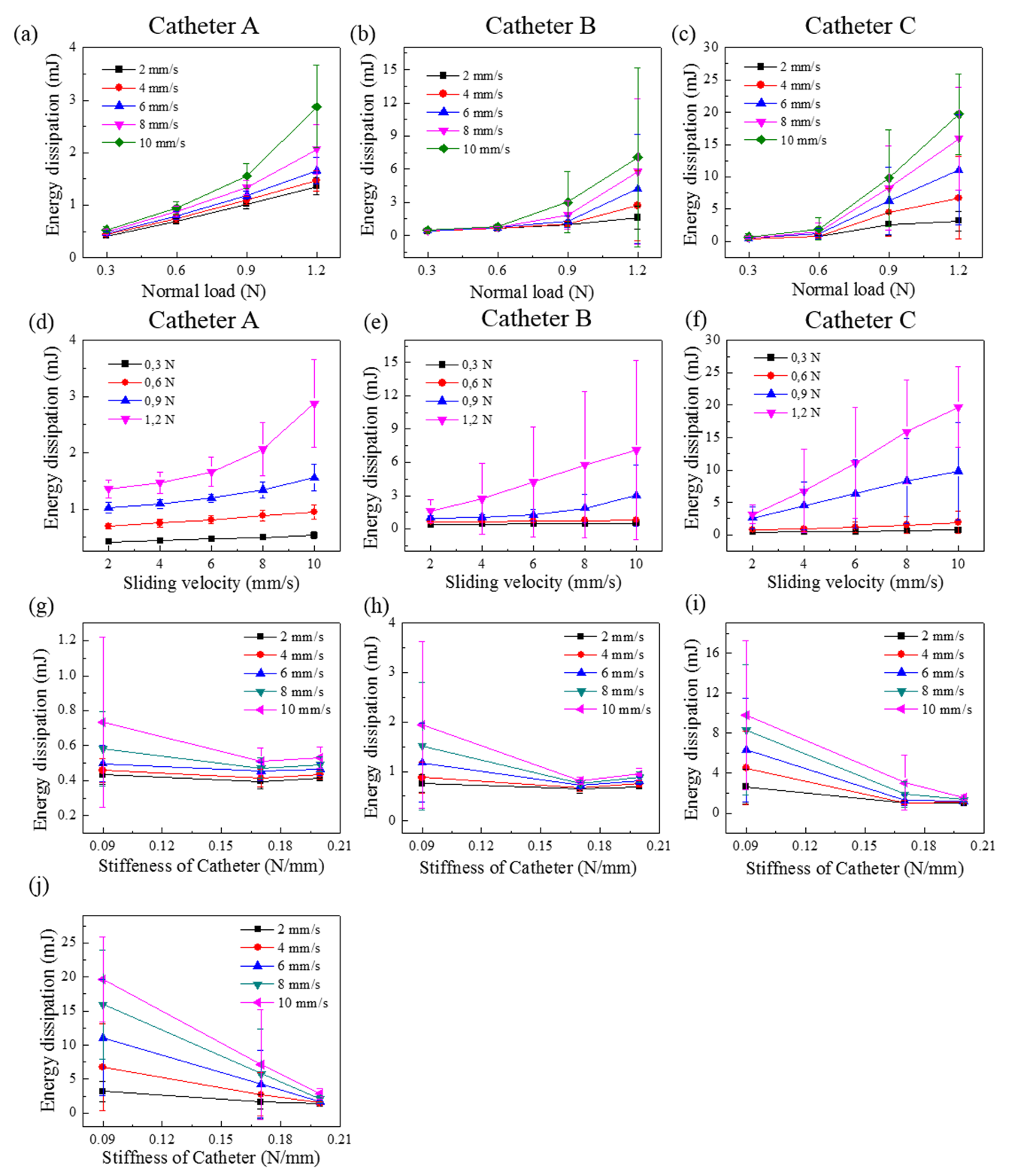


Fig. S3 The frictional energy dissipated for a sliding distance of 30 mm at the catheter-aorta interface under different normal loads (a, b, c), sliding speed (d, e, f) for catheter A, B and C respectively and catheter stiffness at different normal loads (g: 0.3 N, h: 0.6 N, i: 0.9 N, j: 1.2 N).

**3.6 The energy dissipation at the aorta-catheter interface**

Since the two sliding surfaces displace under the action of lateral friction force a certain amount of work needs to be done to overcome the frictional energy which gets dissipated [1]. Typical frictional force versus displacement (*Ft–D*) curve in a reciprocating friction cycle was shown in Fig. S2. The curves in Fig. S2 were randomly ninth cycle of all curves. The area inside each closed graph is the energy dissipated in each reciprocating friction cycle. Complete results of cumulative energy dissipated at the catheter-aorta interface at variable normal load, sliding velocity and catheter loop stiffness are presented in Fig. S3. The energy dissipation increases sharply due to the increasing friction force. Besides, the curve presents a stick-slip phenomenon with the increasing normal load, especially the curve in Fig. S2 d.

The energy dissipation has a direct correlation with the damage of the soft tissue [2]. With the increasing normal load and sliding velocity, the energy dissipation also increases according to Fig. S3 (p<0.05). The energy dissipation has little variation when the normal load changes from 0.3 N to 0.6 N for catheter B and C. Similar to the tendency of COF, the value of energy dissipation also shows an order of magnitude difference from catheter A to C because of difference in stiffness. For higher normal load and greater sliding velocity, the friction force-displacement curve changes dramatically due to the unstable contact between the catheter and aorta. From Fig. S3 g-j, the value of energy dissipation significantly increase. The energy dissipation was the product of friction force and sliding distance, while the COF was the ratio between the friction force and normal load. As a result, the error bars in Fig. S1 and Fig. S3 are of different magnitude.


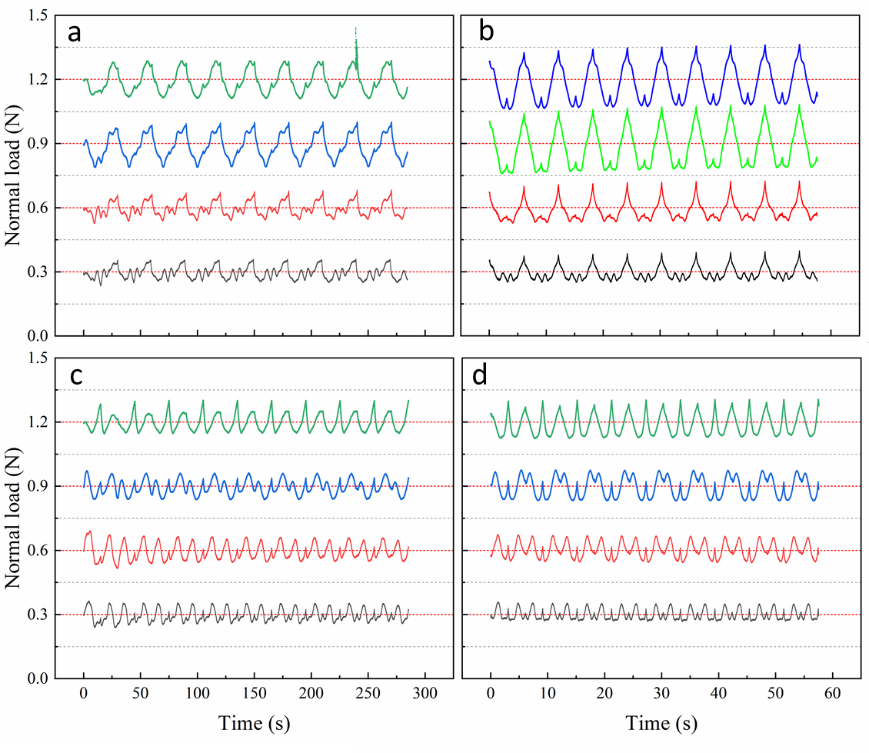


**Figure S4**. Profiles of normal force for 10 cycles from four different friction measurements at 2 mm/s (a, c) and at 10 mm/s (b, d). Fluctuations in normal force are still present despite the UMT-3 carriage (up-down) movement and pinning aorta on silicone rubber. Coefficient of friction was calculated by dividing the measured friction force by the applied normal force at each time point.

[1] C.X. Lin, · W Li, · H Y Deng, · K Li, · Z R Zhou, 67 (2019) 9. https://doi.org/10.1007/s11249-018-1123-x.

[2] W. Li, L. Shi, H. Deng, Z. Zhou, Tribol. Lett. 55 (2014) 261–270. https://doi.org/10.1007/s11249-014-0356-6.
